# Sex differences in neuromuscular androgen receptor expression and sociosexual behavior in a sex changing fish

**DOI:** 10.1371/journal.pone.0177711

**Published:** 2017-05-16

**Authors:** Eric R. Schuppe, Devaleena S. Pradhan, Kevin Thonkulpitak, Cathleen Drilling, Michael Black, Matthew S. Grober

**Affiliations:** 1 Department of Biology, Georgia State University, Atlanta, Georgia, United States of America; 2 Neuroscience Institute, Georgia State University, Atlanta, Georgia; University of Missouri Columbia, UNITED STATES

## Abstract

Androgen signaling, via receptor binding, is critical for regulating the physiological and morphological foundations of male-typical reproductive behavior in vertebrates. Muscles essential for male courtship behavior and copulation are highly sensitive to androgens. Differences in the distribution and density of the androgen receptor (AR) are important for maintaining dimorphic musculature and thus may provide for anatomical identification of sexually selected traits. In *Lythrypnus dalli*, a bi-directional hermaphroditic teleost fish, both sexes produce agonistic approach displays, but reproductive behavior is sexually dimorphic. The male-specific courtship behavior is characterized by rapid jerky movements (involving dorsal fin erection) towards a female or around their nest. Activation of the supracarinalis muscle is involved in dorsal fin contributions to both agonistic and sociosexual behavior in other fishes, suggesting that differences in goby sexual behavior may be reflected in sexual dimorphism in AR signaling in this muscle. We examined sex differences in the local distribution of AR in supracarinalis muscle and spinal cord. Our results demonstrate that males do express more AR in the supracarinalis muscle relative to females, but there was no sex difference in the number of spinal motoneurons expressing AR. Interestingly, AR expression in the supracarinalis muscle was also related to rates of sociosexual behavior in males, providing evidence that sexual selection may influence muscle androgenic sensitivity to enhance display vigor. Sex differences in the distribution and number of cells expressing AR in the supracarinalis muscle may underlie the expression of dimorphic behaviors in *L*. *dalli*.

## Introduction

The expression of male-typical reproductive behavior in vertebrates is often mediated by androgen signaling through the androgen receptor (AR) to activate both central and peripheral neural circuits that innervate sexually dimorphic muscle targets [1, 2]. As a result, males often express more AR in the dimorphic muscles necessary for their displays, as well as the regions of the spinal cord that innervate these tissues [3–6]. Moreover, emerging work suggests that neuro-motor androgenic sensitivity may be an important substrate on which selection acts to enhance the fine-motor behavior used in male courtship [7, 8]. As a result, individual or sex differences in muscle and spinal cord AR expression may be related to courtship display performance. To date, little research has investigated this idea outside of avian species [7, 9], leaving a gap in our knowledge regarding the role of local androgen signaling in the production of adaptive courtship displays.

Peripheral activation of muscle AR is necessary for the production of sexually selected display maneuvers in avian species [3]. Similarly, in fish, AR expression is often wide-spread in muscles that underlie the production of sociosexual displays. Activation of AR via the potent fish androgen 11-ketotestosterone (KT) can have dramatic effects on the expression of territorial and courtship behaviors [10–12]. For instance, in plainfin midshipman fish (*Porichthys notatus*), AR expression is abundant in the sexually dimorphic vocal muscle of males and has been suggested to modulate aspects of muscle performance [13]. Aside from androgenic signaling in muscle, ample evidence suggests that elevated AR expression in spinal motor circuits that directly underlie reproductive behavior is necessary to facilitate these motor acts [7, 14–16]. For instance, androgenic signaling has neuro-protective effects on motoneurons, and blocking such signaling alters characteristics of motoneuron morphology that may diminish an animal’s capacity to produce reproductive behaviors [17–19]. Given these observations, it is possible that elevated expression of AR in muscles and spinal circuits involved in courtship display may co-vary with courtship display rates in other fish species.

*Lythrypnus dalli*, a bi-directionally hermaphroditic teleost fish, lives in small harems of one dominant male and multiple subordinate females. Reproductive behavior is sexually dimorphic, such that only males establish and aggressively defend their nest and court females [20, 21]. ‘Jerk’ swims, the male-specific courtship behavior, are characterized by rapid jerky movements, during which both the pelvic and dorsal fins are extended and retracted [22]. In contrast to the jerk behavior, agonistic ‘approach’ behavior, which is characterized by rapidly accelerating towards and stopping within two body lengths of a conspecific, is readily displayed by both sexes [20]. Activation of the supracarinalis muscle, a muscle group located on the dorsal surface that spans from the beginning of the dorsal fin to the caudal peduncle, is involved in fin shape modification [23, 24]. Activation of the posterior supracarinalis, along with caudal fin muscles, aid in many maneuvers including braking, kicking, and gliding, by rapidly changing the shape of the dorsal and caudal fins [23, 25]. In sum, these studies suggest that the supracarinalis is a key muscle group involved in the expression of social behaviors, including approaches and jerk swims in *L*. *dalli*, which are vital to reproductive success.

The current study investigates whether expression of AR is sexually dimorphic in supracarinalis muscle and spinal cord, relative to epaxial/hypaxial muscles used for general swimming. We expect that this muscle will be hypertrophied in males, because they utilize this muscle more frequently to produce both courtship and agonistic behaviors. Since AR is necessary to maintain aspects of male phenotype in the absence of dimorphic levels of KT and facilitates adult phenotypic transitions [26–28], elevated androgenic sensitivity may similarly maintain musculature and promote the expression of male-typical behavior. If the supracarinalis muscle is required for male-typical sexual behavior (jerking), then we expect that there will be pronounced sex differences in AR in this muscle and perhaps in associated areas of the spinal cord, where motoneurons that drive this muscle are located. In contrast, AR expression would be elevated in both sexes in muscles that serve social behavior used by both sexes (e.g, aggression), and would be low and not sexually dimorphic in muscles that do not contribute specifically to social behavior (e.g. epaxial / hypaxial swimming musculature). Given the importance of activating this muscle for sociosexual display behavior we also predict that local levels of AR protein expression could be associated with display vigor.

## Methods

### Animals

Male (n = 12) and female (n = 36) *L*. *dalli* were collected in June and July 2001 near the Wrigley Institute of Environmental Studies (University of Southern California), Santa Catalina Island, California (33°26'40.8"N 118°29'00.9"W) We made all collections by scuba diving and use of hand nets. Animals were transported back to a laboratory at the USC Wrigley Institute for Environmental Science where they were placed into 12 unique social groups consisting of one male and three females, which were at least 3mm smaller than males. All social groups were constructed on approximately the same date and no individual fish was used in multiple social groups. Each group was maintained in a 40 L aquarium containing a PVC nesting tube. Tanks were provided with a constant supply of Pacific Ocean water at 18.3 C, exposed to the natural light-dark cycle, and fish were fed frozen brine shrimp twice daily. For each fish, we measured standard length, body weight (g), and genital papilla length to width ratio. At the end of the study, all males (n = 12) and random subset of females (n = 8) from different social groups were then euthanized by exposure to excess tricaine methanesulfonate (MS-222; 0.5 mg/100 mL H_2_O), and sex was confirmed by examining gonad and genital morphology [29]. All research was carried out in accordance with Institutional Animal Care and Use Committee at the University of Southern California (IACUC protocol #10262). Capture of field collected animals was approved by CA Department of Fish and Game (Permit #: 803034–01).

### Behavioral observations

To allow for the formation of stable social groups and thereby minimize the effects of capture-induced stress on behavior and physiology, fish groups were housed together for three weeks prior to behavioral observations. Previous work has established that 3–5 days is a sufficient amount of time to form a stable social group in this species. We observed male behavior in these groups five times over the course of three days. In total, we had three morning (08–10:00 h) and two afternoon (14–16:00 h) observation periods. All behavioral observations lasted for 10 min per group. During these observation periods, we totaled the number of approach bouts (agonistic) and jerk swims (courtship) produced by the focal male and directed at females within the social group. The total amount of behavior produced by each individual was divided by observation length to produce approach or jerk rate per minute. Following the last morning observation period, animals were rapidly sacrificed as described above.

### Immunohistochemistry

**Fig 1:**
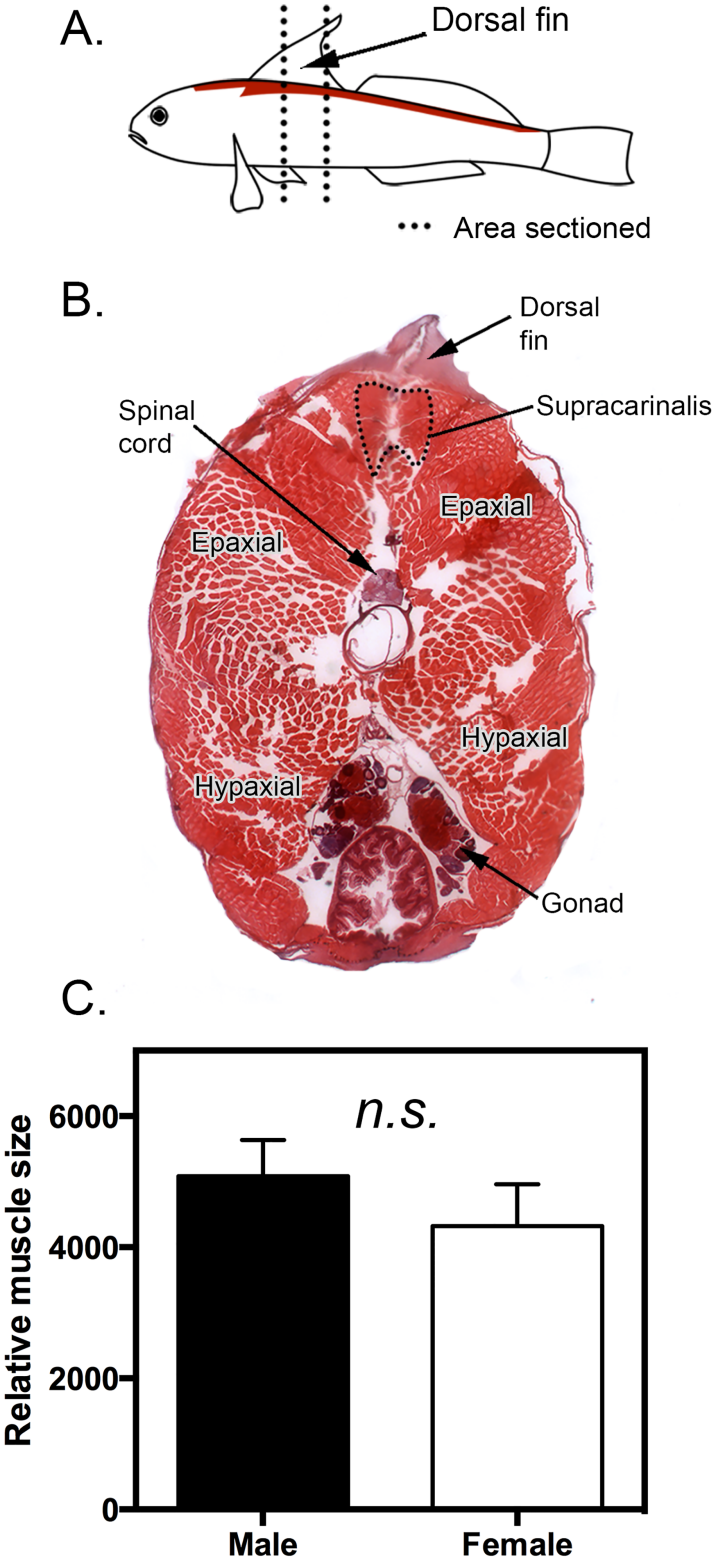
Location and size of the supracarinalis muscle in *L*. *dalli*. Illustration of where the supracarinalis muscle is located (shown in red) and relative area sectioned in this study to assess receptor expression (**A**). Hemotoxylin and eosin stained cross section showing the exact location of the supracarinalis muscle relative to other muscles and peripheral tissues (**B**). Mean (±SEM) relative supracarinalis muscles size (muscle area (μm^2^) / standard length (mm) in male (n = 10) and female (n = 7) *L*. *dalli* (**C**).

Fish were immersed in Bouin’s fixative for 7 days, rinsed in 50% ethanol, and stored in ethanol until processed. Fish were then embedded in paraffin (46–48 C melting point) and sectioned at 20 μm. Since we were primarily interested in the role the supercarinalis muscle in the control of the dorsal fin movement, we intentionally chose sections that contained this muscle (see Fig 1A/1B).

Sections were deparaffinized in Citrasolv and rehydrated through an ethanol-water series. After washing hydrated slides in dH_2_0, antigen retrieval was performed with citric acid buffer (10mM citric acid, 0.05% Tween 20, pH 6.0) as described in Muchrath and Hoffmann [30]. Following antigen retrieval, sections were washed in 0.1M phosphate buffer (PB, pH 7.4) twice for 7 minutes each. Sections were then incubated in a blocking solution (normal goat serum and 0.2% Triton-X in 0.1M PB) for 20 minutes. Next, the sections were incubated in a rabbit polyclonal primary antibody, AR (PG-21, Millipore) diluted (1/250) in normal goat serum and 0.2% Triton-X in 0.1M PB overnight at 4 C. This AR antibody has been widely used in other teleost fish species [30–33] and has been previously validated for *L*. *dalli* [26]. Biotinylated anti-rabbit secondary antibody (KPL) was added to the slides for 30 minutes. The sections were then washed twice in 0.1M PB for 7 minutes each and then incubated in streptavidin-peroxidase (KPL) for 30 minutes. AR positive cells were visualized using 3,3’-diaminobenzidine (DAB) (Sigma Chemical). The DAB solution was prepared using SigmaFast^™^ DAB tablets in accordance with the manufacture’s instructions (Sigma Chemical). The sections were then dehydrated in an ethanol series, cleared in Citrasolv, and mounted with permount. As a control, sections (n = 2 per sex) were processed in a similar manner, except for the omission of the primary antibody. Expression of gonadal AR was used as a positive control on sections where muscle and spinal cord expression was being evaluated.

### Image analysis

Images were acquired using a Zeiss Axioplan microscope and Axiovision image capture software (v. 4.1). For each fish, one section at approximately the same point within the body was selected. In this randomly chosen section, supracarinalis area was measured by tracing the outside of this muscle group. Given the pronounced size differences between males and females in this species, muscle area was standardized to the standard length of each individual (muscle area (μm^2^) / standard length (mm)). In addition, two independent observers who were blind to the sex of the animal counted all AR positive supracarinalis muscle cells in each section. The number of AR cells was also counted within both the dorsal and ventral horns of the spinal cord. Additionally, the number of AR cells in swimming muscle was used as a control, as these muscles do not contribute to rapid starting and stopping or dorsal fin flexion. To perform these counts in epaxial and hypaxial muscles, we randomly chose a 400x300μm area of musculature and counted all AR+ cells within this area. This 120,000 μm^2^ area approximates the average area of the supracarinalis muscle (both males and females combined, 156,009.8 ± 17,533.98). All cell counts and measurements were performed using ImageJ (National Institute for Health) on the previously acquired digital images.

### Statistical analyses

Two-way analysis of variance (ANOVA) was used to examine the effect of sex and muscle type (supracarinalis, epaxial, hypaxial) on the number of AR positive cells. Similar two-way ANOVAs were used also to examine differences in number of AR cells in the dorsal and ventral horns of the spinal cord. When applicable, *post hoc* analyses were used to further examine differences. These analyses were corrected using the Benjamini & Hochberg method to account multiple comparisons. Linear regression analyses were used to examine the relationship between jerk and approach rate and the number of AR expressing cells in each tissue. Data that were not normally distributed, as indicated by Q-Q plots and Shapiro-Wilk tests, were log transformed. All analyses were carried out using R. Significance was set at α = 0.05. All data are represented as mean ± SEM. Data used to perform these analyses can be found in the supplemental information.

## Results

### Supracarinalis muscle

Relative muscle size was not significantly different between males and females, indicating that this muscle is not hypertrophied in males (t_15_ = 0.91, p = 0.38; Fig 1C).

**Fig 2:**
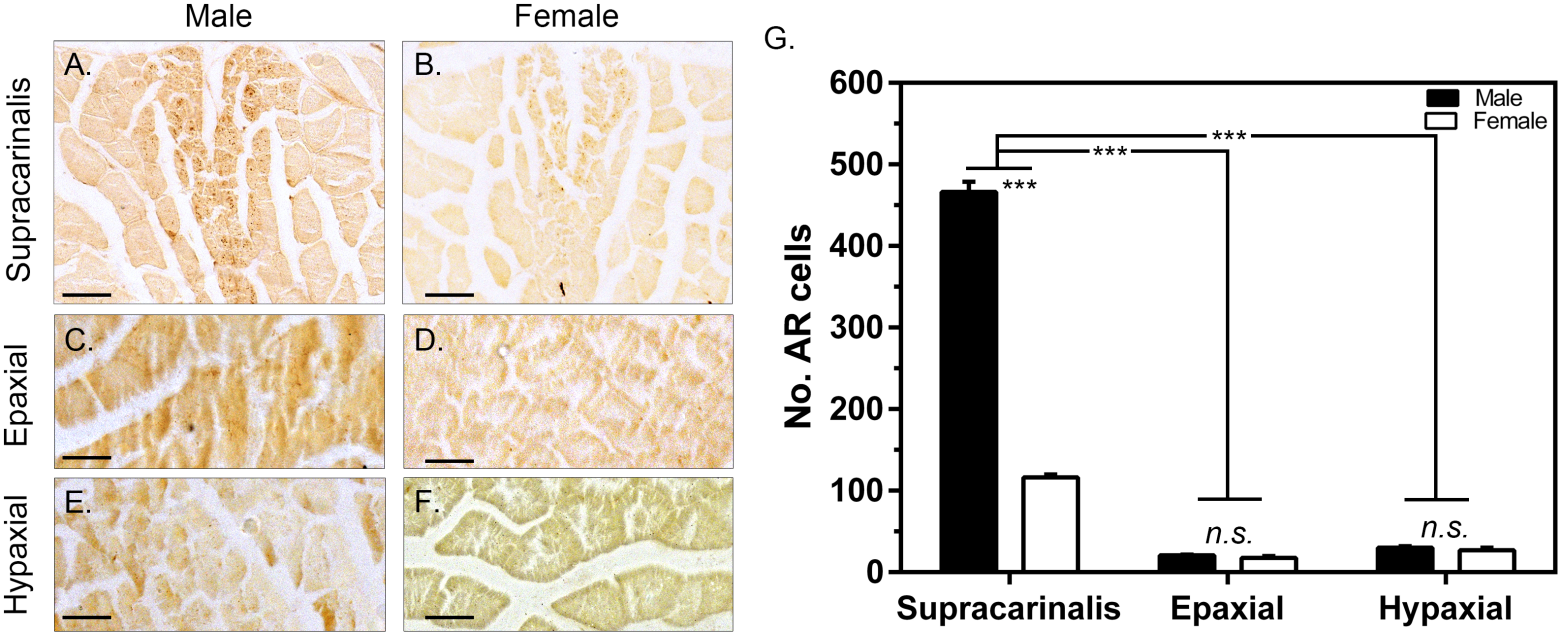
Immunolocalization of androgen receptor (AR) expression between sexes in different muscle types. Representative AR staining within the supracarinalis muscle of males (**A**) and females (**B**), and the epaxial and hypaxial muscles of males (n = 12) (**C/E**) and females (n = 8) (**D/F**) respectively. The mean (±SEM) number of AR positive cells within the supracarinalis, epaxial, and hypaxial muscles in males and females (**G**). All images were captured under a 40x objective. Scale bar = 50 μm *** denotes a significant difference at p < 0.001.

Androgen receptor expression was significantly different between muscle types (F_2,54_ = 17.02, p <0.001; Fig 2) and sexes (F_1,54_ = 13.58, p <0.0001; Fig 2). Furthermore, there was an interaction between muscle type and sex (F_2,54_ = 3.68, p = 0.03; Fig 2). AR expression within the supracarialis muscle was significantly higher than both epaxial (p <0.01) and hypaxial (p <0.01) musculature. In addition, *post hoc* analyses showed that males exhibited significantly greater AR expression compared to females in the supracarinalis, but not the other muscles examined.

### Spinal cord

**Fig 3:**
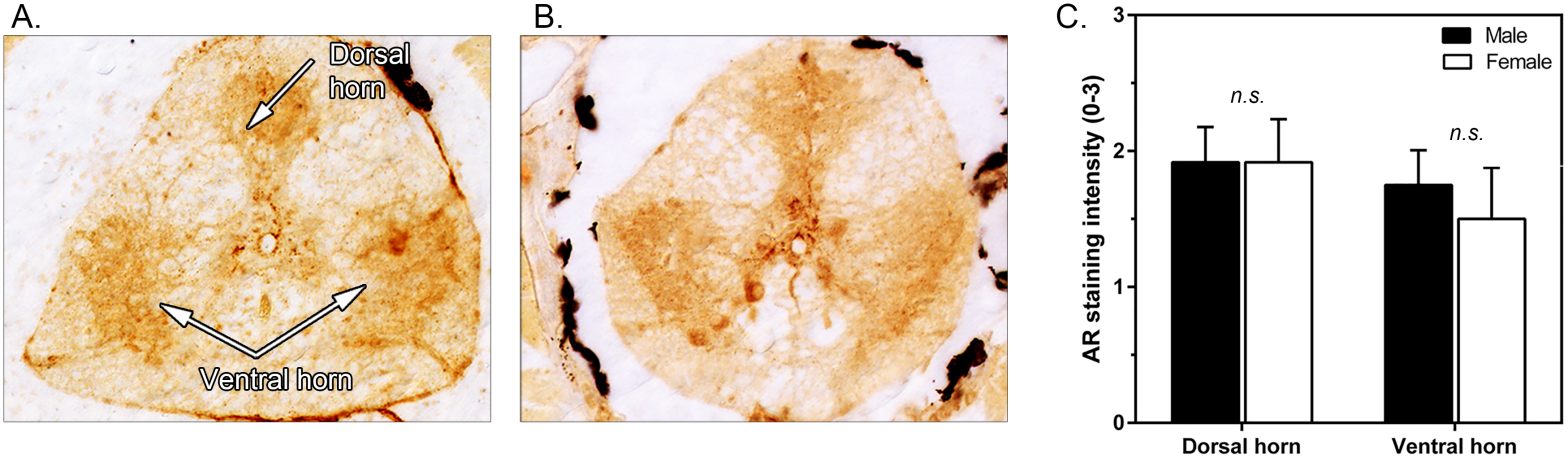
Immunolocalization of spinal cord androgen receptor (AR) expression between the sexes and differences in expression between the sexes in the ventral and dorsal horn of the spinal cord. Representative AR staining in the dorsal and ventral horn of male (n = 12) (**A**) and female (n = 8) (**B**) *L*. *dalli*. Mean (±SEM) AR staining intensity within the dorsal and ventral horns of the spinal cord in males and females (**C**). DH = dorsal horn, VH = ventral horns.

Male and female *L*. *dalli* exhibited comparable levels of AR expression within the spinal cord (F_1,36_ = 0.35, p = 0.56). Additionally, there were no differences in AR expression within the ventral or dorsal horns of the spinal cord (F_1,36_ = 0.01, p = 0.99; Fig 3).

### Muscle-behavior association

**Fig 4:**
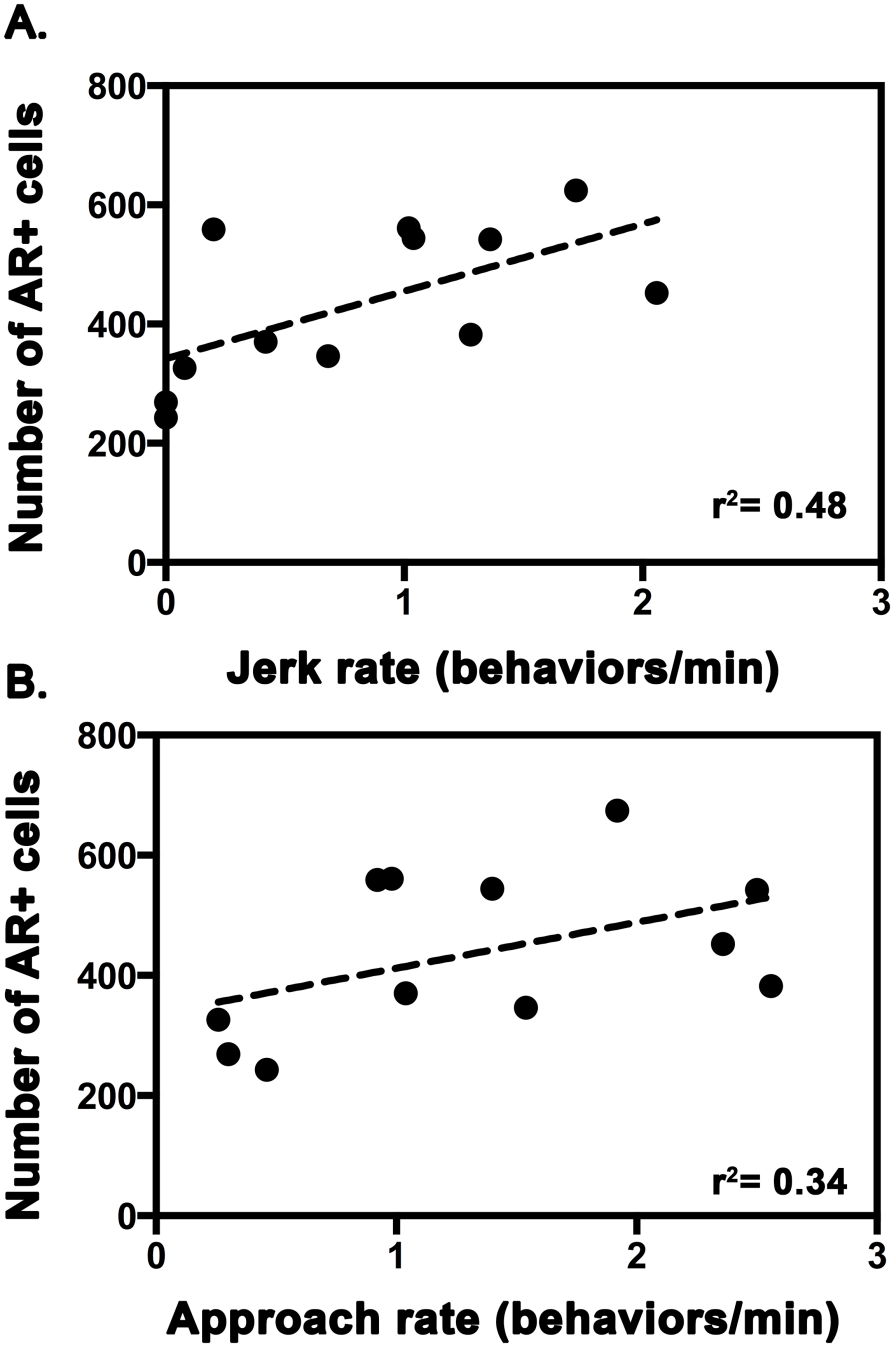
The relationship between the number of androgen receptor (AR) expressing cells in the supracarinalis muscle and the rate of jerk (A) and approach (B) behaviors in male *L*. *dalli* (n = 12).

There was a positive association between jerk rate and AR expression within the supracarinalis muscle (Fig 4, r^2^ = 0.48, F_1,10_ = 9.09, p = 0.013). Similarly, males that approached other fishes more exhibited grater levels of AR expression within the supracarinalis (Fig 4, r^2^ = 0.34, F_1,10_ = 5.18, p = 0.046).

## Discussion

In the current study, we demonstrate that AR expression is widespread and sexually dimorphic in neuromuscular tissues that coordinate social signaling in *L*. *dalli*. Furthermore, we demonstrate for that higher muscular AR expression is associated with greater rates of courtship and aggressive displays. Thus, our findings provide support for the hypothesis that differences in androgen sensitivity are likely to be crucial in modulating social behaviors that have important fitness consequences. Contrary to our prediction, AR expression was not sexually dimorphic within any region of the spinal cord. Thus, we suggest that selective elevation of androgen sensitivity in muscles that underlie sociosexual signaling may augment muscular capabilities to allow animals to display at higher rates. Consistent with previous findings [26], we suggest that local changes in AR expression may be one mechanism by which *L*. *dalli* exhibits rapid shifts in behavior during adult phenotypic transitions [26].

### Supracarinalis muscle and sociosexual behavior

While we predicted that there would be pronounced sex differences in the size of the supracarinalis muscle, male and female muscles are not hypertrophied relative to the body musculature and are comparable in relative size. In other vertebrates, muscles that control male-typical reproductive behaviors are often dramatically larger in both size and weight [34–37]. One explanation for this finding is based on the life history of this organism. *Lythrypnus dalli* is a bi-directionally hermaphroditic fish that can undergo rapid adult sex change, wherein fishes can transform reproductive tissue morphology and begin to reproduce as the opposite sex in under two weeks [38]. More importantly, females can display male-typical courtship jerks within minutes of beginning this phenotypic transition [39]. Consequently, it would be necessary for females to maintain muscle morphology that allows them to quickly produce jerk swims if the opportunity arose. Another aspect that may contribute to the lack of dimorphism is that both sexes activate this muscle group to rapidly accelerate and brake when producing agonistic behaviors, including approaches.

Sexually dimorphic AR expression may play a critical role in both masculinizing neuro-motor circuits and maintaining optimal muscle function to thereby regulate the ability to produce social signals. Since this species exhibits no sex differences in levels of circulating androgens [27, 40, 41] or supracarinalis muscle size (Fig 1), local androgen sensitivity may maintain dimorphic reproductive behavior. Previous work in *L*. *dalli* shows that differences in AR expression are vital to maintain other sexually dimorphic reproductive traits, and blocking androgenic signaling de-masculinizes such traits in adult males [26]. Given this, we suggest that an up-regulation in AR expression in this muscle may similarly promote the expression male-typical traits, including behavior, during female-to-male sex change. Such changes would allow females undergoing adult phenotypic transitions to co-opt muscles for new purposes, without the energetically costly effects of rapidly transforming muscle structure. Furthermore, shifts in receptor density as opposite to modifying muscle structure may permit animals to express behavior more quickly. Since social status governs adult phenotype in *L*. *dalli*, we contend that rapid changes in AR expression may gate reproductive behavior, and thereby be an additional mechanism to promote sex change in either direction. Taken together, we suggest that differential AR expression plays an important role in maintaining sexually dimorphic phenotype and behavior in sexually plastic vertebrates.

Along these lines, our findings also indicate that there may be a threshold of AR expression that males must maintain in the supracarinalis muscle to express different types of sociosexual behaviors. For instance, male *L*. *dalli* have co-opted this muscle to produce both agonistic and courtship displays, whereas females only exhibit the agonistic approach behaviors. Given this, the low levels of AR expression in females is likely important in modulating approach behaviors used by both sexes, but this lower expression relative to males may functionally prohibit them from producing the male-typical courtship display.

A rich literature on a diverse assemblage of vertebrate species demonstrates that males often maintain greater levels of androgen receptor expression in tissues that underlie the expression of male-typical traits, including courtship behavior [4, 7]. We add to this literature by demonstrating that expression of AR is sexually dimorphic in a muscle that may coordinate the production of courtship and agonistic behavior in *L*. *dalli*. Interestingly, both males and females in this species maintain higher AR expression in the supracarinalis muscle relative to muscles that are not specifically involved in the production of social behaviors (Fig 2). Yet, levels of AR expression in the male supracarinalis muscle are ~4.5x higher than in females. Therefore, we suggest that differences in androgen sensitivity modulate the ability to produce courtship and agonistic displays more frequently. This idea is supported by research that demonstrates muscles that coordinate sociosexual behaviors, including both courtship and agonistic displays, frequently exhibit increased AR expression [16]. Such elevated androgenic sensitivity likely enhances motor ability by regulating molecular factors that regulate contraction speed and stamina [14, 42–44]. Accordingly, androgenic modulation of suites of genes that alter motor performance may explain the differences in display rate observed in the current study. Nevertheless, we cannot rule out the possibility that certain individuals are predisposed to having more AR in this muscle or that repeated use of the muscle increases AR expression. Investigations of the effects of exercise on muscle AR content have often found conflicting results, with some studies showing repeated exercises increases muscle AR expression [45, 46] while others show training has no effect [47, 48].

The current study and others [4, 9], provide evidence of multiple avenues by which androgenic signaling processes may modulate sociosexual behavior. In green anoles (*Anolis carolinensis*), male courtship behavior is associated with high circulating levels of testosterone, but not elevated local AR expression [9]. While the levels of circulating androgens were not assessed in the current study, previous work in *L*. *dalli* has demonstrated that both sexes maintain low and comparable androgen levels [27, 40]. Thus, local differences in receptor expression may play a critical role in modulating reproductive behavior in *L*. *dalli*. Nevertheless, these two examples highlight the idea that behavioral flexibility can be attained via modulation of different aspects of androgenic signaling. To augment display performance, selection may act on local androgenic sensitivity and/or by mechanisms that regulate circulating levels of androgens.

### Androgenic sensitivity in spinal cord

While AR is abundantly expressed within the spinal cord of *L*. *dalli*, we did not observe any sex differences in expression. Although this result does not support our prediction, work in other vertebrates, investigating sex differences in AR expression in regions of the spinal cord that innervate the muscles responsible for controlling aspects of reproductive behavior, often finds contradictory results [13, 14, 49, 50]. For instance, in frogs and rodents males frequently exhibit sexually dimorphic AR expression in spinal circuits that coordinate reproductive behaviors [50], while work on golden-collared manakins, a species that performs elaborate and physically complex displays, did not find sex differences in AR in the spinal cord [14]. Instead, work in this system suggests that sex differences in local steroidogenic enzyme expression may contribute to sex differences in display behavior [51]. We similarly expect that differences in androgenic biosynthesis in the spinal cord may contribute to sexually dimorphic behavior. In hermaphroditic species like *L*. *dalli*, a lack of sex differences in circulating androgens, coupled with rapid changes in local androgen biosynthesis might be important mechanism for exhibiting opposite sex reproductive behaviors during adult phenotypic transitions.

### Conclusions

In the current study, we show clear evidence that AR expression is sexually dimorphic and elevated in muscles that control sociosexual behavior. Additionally, individual differences in AR expression in the supracarinalis muscle are associated with differences in male display rates in *L*. *dalli*. Thus, we suggest that differences in androgenic sensitivity may augment display vigor in this species. Given that display vigor is thought to be important during assessment of mate quality, we expect that local differences in AR sensitivity is a response to sexual selection.

## Supporting information

S1 File Data on tissue specific AR expression and behavioral measures. Column labeled ‘ID’ is the individual fish for which behavior and AR expression was collected. The ‘muscletype’ column includes the supracarinalis and control muscles (e.g. epaxial and hypaxial). The ‘SCarea’ column the two spinal cord that were assessed (dorsal/ventral horn).
(XLS)
